# Research contributions on childhood obesity from a public-private partnership

**DOI:** 10.1186/1479-5868-12-S1-S1

**Published:** 2015-07-27

**Authors:** Cheryl L Perry, Deanna M Hoelscher, Harold W Kohl III

**Affiliations:** 1 Michael & Susan Dell Center for Healthy Living, The University of Texas School of Public Health, Austin Regional Campus, Austin, TX, USA

**Keywords:** child, adolescent, parent, schools, obesity, partnership, theory, cultural, intervention, socioeconomic inequalities

## Abstract

**Background:**

Childhood obesity remains a significant global problem with immediate and long-term individual health and societal consequences. Targets for change should include the most potent and predictive factors for obesity at all levels of the personal, social and physical environments. The Michael & Susan Dell Center for Healthy Living (‘the Center’) is a public-private partnership that was developed to address child health issues through research, service, and education. This overview paper introduces a special issue of seven articles on childhood obesity from the Center, and the implications of this research for obesity prevention.

**Methods and results:**

A review of the literature on public-private partnerships was undertaken and key components of the partnership between the Michael & Susan Dell Foundation and the Center were compared for compatibility. The conceptual framework for Center research, based on social cognitive theory and the social-ecological model, is explained. An overview of papers in this special issue, relative to the conceptual framework, and the implications of this research for childhood obesity prevention, are provided.

**Conclusions:**

The public-private partnership that created the Michael & Susan Dell Center for Healthy Living has been instrumental in motivating the Center’s academic faculty to focus their research on improvements in child, family and community health through etiologic, epidemiologic, methodologic and intervention research. This special issue extends this work and places particular emphasis on socioeconomic inequalities in addressing the obesity problem in the U.S. and worldwide.

## Background

Although numerous studies have addressed both causes and solutions to the childhood obesity epidemic, much remains to be discerned. The purpose of this special issue of the *International Journal of Behavioral Nutrition and Physical Activity* is to not only further our understanding of the health consequences, epidemiology, etiological factors, and effective programs and policies that are relevant to the epidemic, but also to provide resources and succinct, evidence-based, comprehensive, and instructive knowledge for future research and practice. Ongoing updates of the knowledge base of the childhood obesity epidemic are important and urgent due to the rapid increase in the prevalence of obesity in both developed and developing countries during the last 30–40 years, despite countless initiatives and research to address childhood obesity. Although this topic has received considerable attention, substantive gaps in the literature remain. Each of the papers in this special issue extends this knowledge with results of ongoing research and through updated reviews of existing work.

Although anthropologic data suggest that humans have been steadily gaining weight for more than 100 years [1], obesity has become a dominant health concern over the past 30–40 years in the United States and other developed countries. Early in the 20^th^ century, the primary problems related to body weight were insufficient nutrition and low caloric intake. Current standards indicate a healthy body mass index (BMI= weight(kg)/height^2^(m)) for adults is 18 to <25 [2]. In children, obesity is defined as having a BMI above the 95^th^ percentile of the BMI norm-referenced and age-specific growth charts [3]. Starting around 1980, a notable increase in the prevalence of obesity (>30 BMI in adults) was observed, and this increase has continued to occur in both adults and children worldwide [4].

Obesity is associated with substantial health-related problems, including those related to physical, psychological, and social health, as discussed in other papers in this special issue (see Carey et al. paper in this issue on educational attainment and social health, for example [5]). Overall, the relative risk of premature death begins to increase when BMI is above 30. Because longitudinal data suggest that obesity appears to track from childhood to adulthood, early prevention and intervention efforts are critical beginning early in life [6].

These efforts should be guided by identifying and modifying the most potent behavioral, environmental, and psycho-social risk factors for childhood obesity, from proximal factors such as adequate knowledge and skills, to more distal factors, such as policies that mandate healthful foods or physical education in schools. Our goal is to cover a range of concerns related to childhood obesity and present new data and solutions for consideration.

The setting for our research is the Michael & Susan Dell Center for Healthy Living (‘the Center’) at The University of Texas School of Public Health. The Center is a unique private-public partnership that was established to further scholarship, translation of science into practical applications, and dissemination of evidence-based programs and best practices concerning healthful living among children, families, and communities with a particular focus on childhood obesity. This introductory paper describes this partnership and the conceptual framework guiding our research, and also presents an overview of the ensuing papers in this issue and implications for intervention.

## A public-private partnership for child health

The Michael & Susan Dell Foundation (‘the Foundation’) is a non-profit, 501(c)(3) organization that was founded in 1999 in Austin, Texas. Susan Dell serves as co-founder and Board Chair of the Foundation. The Michael & Susan Dell Center for Healthy Living became a reality with a financial gift from the Foundation to the University of Texas School of Public Health in 2006. (The activities of the Center will be discussed below.) In 2008, Dr. Aliya Hussaini began to lead the Health Team at the Foundation, and under her leadership the health portfolio has been greatly expanded [7]. The Foundation provides the Center with funding for staff, communications, research infrastructure, community outreach efforts, and training opportunities for public health initiatives.

In its most basic form, a public-private partnership is a structured collaboration between private (non-governmental) and public entities [8] toward a common goal. Private entities may seek to engage in these partnerships in order to further their aims in terms of social responsibility and the expectations of their stakeholders. Public entities can learn new skills though these partnerships, obtain additional funding, and extend their reach with regard to their basic mission [8]. Kraak & Story [9] provide multiple examples of public-private partnerships that support healthy lifestyles to reduce childhood obesity. For example, the General Mills Foundation partnered with both the American Dietetic Association Foundation (now the Academy of Nutrition and Dietetics Foundation) and the President’s Council on Physical Fitness and Sports (now the President’s Council on Fitness, Sports & Nutrition), to provide small grants, called Champions for Healthy Kids, to community organizations with the goal of increasing physical activity [9].

The World Health Organization also participates in multiple public-private partnerships “to achieve a health-creating goal on the basis of mutually agreed and explicitly defined division of labor” (p. 748) [10]. The Robert Wood Johnson Foundation has a strong program in public health and partnerships with public entities throughout the U.S. [11]. Thus, while there is some controversy over whether researchers who are working to decrease childhood obesity should partner with certain private entities, such as food companies [12], creating public-private partnerships may have value if the partners are compatible in their mission, target audience, cause, and evaluation methods. Dimensions of compatibility between private and public entities are discussed in Kraak & Story [9]. These dimensions—essentially the qualities and characteristics of an organization (e.g., mission)—are shown in Table 1 with regard to the Michael & Susan Dell Foundation and the Michael & Susan Dell Center for Healthy Living, and illustrate a high degree of compatibility between these entities.

**Table 1:** Dimensions of Compatibility between the Michael & Susan Dell Foundation (“Foundation”) and the Michael & Susan Dell Center for Healthy Living (“Center”) as adapted from Kraak & Story [9]

**Dimension of Compatibility**	**Foundation**	**Center**
1. Mission	Improving the lives of children living in poverty	Healthy children in a healthy world
2. Resources	Financial, business resources	Researchers and students, grant funding
3. Management	Health leader with MD, MPH and trained in public policy	Executive Committee with training in nutrition, physical activity, kinesiology, and behavioral sciences
4. Workforce	Strong worksite health promotion program	Center is housed in School of Public Health with faculty and students who have a health promotion orientation
5. Target market	Low income and underserved youth	Youth and their parents, schools, and communities, with a focus on economically disadvantaged populations
6. Product	Efforts to reduce childhood obesity	Research on methods to understand and reduce childhood obesity, translation of the research into usable products, dissemination of best practices
7. Cultural fit	Value children’s health world-wide	Value healthy children in healthy environments and communities
8. Evaluation	Milestones are negotiated on a yearly basis	Reports provide details on milestones on a 6-month basis

The Foundation and The University of Texas School of Public Health partnered to create the Center in 2006. Major emphases of the Center have included: (1) the establishment of partnerships and collaborations for prevention and control of childhood obesity throughout Texas and the U.S., such as Live Smart Texas [13,14]; (2) the implementation of annual educational events to recognize and disseminate the latest research on childhood obesity, such as the distinguished Lectureship in Child Health; (3) annual recognition of and dissemination of best practices for community-based obesity prevention through the *Texas Obesity Awareness Week* activities; (4) the provision of support for the piloting, submission and implementation of research related to child health, such as through *pilot-study grants* and staff to support grants management; (5) the establishment of *pre- and post-doctoral fellowships* (Dell Health Scholars) to support and train the next generation of researchers in child health; (6) the review of our Center and mentoring of the faculty through annual meetings of our external *Scientific Advisory Council*, faculty and staff retreats, and grant review sessions; (7) a strong focus on *communications that are relevant to child health* and the Center, by establishing written materials about the Center, a website[15], ongoing blogs, tweets from faculty and staff, webinars, and press coverage; and (8) communications (including infographics) focused on the education of policy makers and health advocates.

### Research at the Michael & Susan Dell Center for Healthy Living

In the area of child health, research at the Center has been guided by two primary conceptual orientations. The first is the social-ecological model, which focuses on the proximal and distal as well as social and physical environments, which affect an individual’s behavior [16]. In this model, the individual is at the center of influence, and intra-personal influences (such as levels of knowledge), social-environmental influences (such as family and peer modeling), the physical environment (such as access to healthy foods), and the policy environment (such as requirements for physical education in schools) progressively and directly influence individual behavior, as well as affect other more proximal influences, which in turn influence behavior. For example, if daily physical education (PE) in schools is mandated at the state level, then that policy will not only influence the physical environment of schools to be able to offer PE to all students, it will also influence the students’ perception of whether daily activity is normative and likely the students’ levels of physical activity as well [17].

The second theoretical orientation is social-cognitive theory (SCT), a key learning theory that provides depth (to the breadth of the social-ecological model) in understanding human behavior as a result of changes in cognitions and environments [18]. Key concepts of SCT are self-efficacy, outcome expectations, and the power of role models [19]. These concepts can be considered across the various influences that are proximal or distal to the individual. For example, young people are influenced *not* to use alcohol when they observe attractive peers who advocate/model against alcohol use [20]. Similarly, role modeling could involve “model” schools or communities that make changes in their environments and influence other schools and communities to do likewise [21-23].

**Figure 1:**
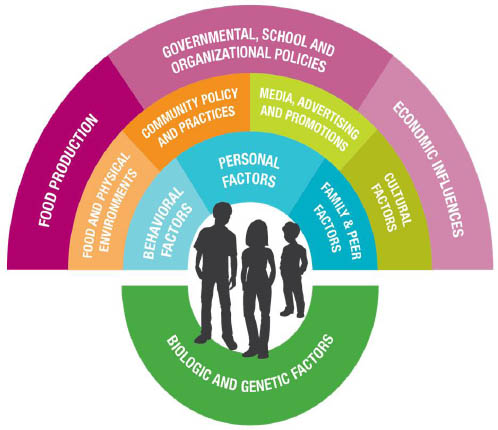
Research Framework for Childhood Obesity from the Michael & Susan Dell Center for Healthy Living.

These two conceptual orientations—the social-ecological model and social cognitive theory—have guided the development of our Center’s overarching research framework for child health (Figure 1). This framework is important because it provides a larger visual organization and model for our research and also serves as a way to characterize research on child obesity, for our Center and for other researchers or organizations. The framework has been particularly useful as we work in various communities to explain the types of interventions and initiatives that may impact childhood obesity. At the center of the framework are the children, adolescents, and specific behaviors that are the foci of our work. The circle most proximal to the children consists of factors most closely related to the behavior of children, including factors that are non-modifiable such as genetic or biological factors, as well as those that can be changed such as personal (cognitive) factors, behavioral factors such as skills and intentions, and family/peer modeling and communications [24]. The second layer in the semi-circle includes those factors that are influential at the school or community levels, such as the food and physical activity environments, community policies and practices, media and marketing, and other cultural influences. The most distal layer includes factors related to children’s behaviors and health at a wider population level, such as statewide or national, including methods of food production, governmental and educational policies, and economic influences. Each of these levels and types of influence are relevant to the problem of childhood obesity.

### Special issue

This supplement continues and expands our Center’s contributions to the science of childhood obesity and its prevention and related risk factors. The articles consist of original scientific work. Each represents an addition to some section or component of the guiding research framework.

At the most proximal level (*biological and genetic factors*), Perez and colleagues examine the reliability and validity of self-reported versus objective height and weight measurements in over 24,000 8^th^- and 11^th^-grade students that are representative of those age groups in Texas [25]. Because of the large sample sizes, and the ethnic/racial diversity of the population, a more robust examination of these measurement methods could be achieved compared to other studies. In addition, the authors suggest correction equations that can be applied to samples when only self-reported data are available, thus improving the validity of BMI measurements in studies with measurement constraints.

*Behavioral factors* are also associated with greater BMI among young people. Carey and colleagues analyzed data from the 2011-2012 National Survey of Children’s Health (n=45,255), for young people ages 10-17 [5]. Children were classified as non-overweight, overweight and obese. Obesity was associated with being absent from school, having problems in school, and not being engaged in school, even when controlling for socioeconomic status and health status. Increased weight, even among tweens and adolescents, is associated with poorer educational outcomes at school, which could have long-term implications.

Ranjit and colleagues examine the *home and neighborhood environments* of 4^th^-grade children in order to tease out whether factors at these levels might be independent of socioeconomic status [26]. They find that both the physical and social aspects of the home environment affect healthy eating, including not watching television during dinner, not eating at restaurants, and availability of healthy foods at the dinner table. These factors are all significantly related to children’s healthy eating, even when controlling for socioeconomic and neighborhood factors.

At a somewhat more *distal level*, Evans and colleagues examine the perceived food environments of low-income communities and potential methods to improve access to healthy foods [27]. Thirteen focus groups were held with a total of 148 residents (80% Hispanic or African American) in low-income communities in order to assess the viability of improved access to supermarkets, farmers’ markets and community gardens, as well as healthier foods in convenience stores, given that the cost of food and easy access were primary barriers. Importantly, the residents expressed preference for a large supermarket – in their neighborhood – in order to best facilitate healthy food choices.

Vandewater and colleagues [28] examined the relationship between being *overweight as children and subsequent television viewing time*, using two waves of data from the Child Development Supplement (CDS) to the Panel Study of Income Dynamics, a nationally representative sample of children and adolescents. They used structural equation modelling to find strong evidence that being overweight in childhood was subsequently associated with time spent watching television, but that this time was mediated by obesity-related social isolation and marginalization in childhood. Thus, while television and obesity may be associated, the social environment in childhood may play a large role in mediating this relationship.

Even more distally, Taber and colleagues examine the impact of *state-level soda bans* on the consumption of soda and other sugar-sweetened beverages (SSBs) [29]. Results show that high school students drink more SSBs, energy drinks, coffee/tea, and sports drinks if they reside in a state that only bans soda from schools and yet have vending machines with other SSBs available at school. Their analyses suggest that states and schools need to have consistent and strict policies on SSBs of all types. *Economic disadvantage* has been shown to be associated with obesity, and Springer and colleagues examine this at the school level, by analyzing the associations between being at a school with a high proportion of students who are economically disadvantaged and BMI among middle school students at those schools [30]. They find that independent of the families’ socioeconomic status, students attending high economically disadvantaged schools were 1.7 to 2.4 times more likely to be obese than students attending schools with a lower percent of economically disadvantaged students. They suggest that structural interventions at schools are needed to address obesity, especially when many of the students are economically disadvantaged.

### Implications for interventions for childhood obesity

Each of the articles in this special issue has important lessons for interventions that may impact childhood obesity. The most salient implications for each of the papers are summarized in Table 2. As a group, the papers also have some common themes. The first is well-recognized: children from lower socioeconomic groups are more likely to be overweight and less likely to have resources (personal or environmental) to facilitate healthier eating and physical activity patterns. But a second theme emerges that is equally important: home, school, and policy environments can serve to alleviate the effects of lower socioeconomic status. These findings provide potential avenues to address overweight and obesity in populations with socioeconomic inequalities, for example, by working with families to improve the home food environment, or to target particular schools for intervention, or to assure consistent policy approaches within schools and communities. A third theme is that the determinants of overweight among young people, and associated interventions, are not one-dimensional. For example, obesity may be associated with television viewing, but there are other factors that mediate this relationship that merit attention, such as supportive social relationships of children, independent of weight status. Also, interventions are likely to be more effective if they address multiple areas or levels of the social environment, as shown in our research framework in Figure 1, rather than just one area, so that parents, schools, communities and policies all support and reinforce healthier food and physical activity choices.

**Table 2:** Implications for Intervention for Childhood Obesity from *IJBNPA* Special Issue Papers

**First Author and Title of Paper [Reference]**	**Implications for Intervention for Childhood Obesity**
**Perez et al.**[25] Measuring the bias, precision, accuracy, and validity of self-reported height and weight in assessing overweight and obesity status among adolescents using a surveillance system.	Although there are race/ethnicity and gender differences, young people ages 12-18 can fairly accurately self-report their heights and weights. This may be useful with working with adolescents who are overweight and obese, to help them set goals that are relevant to their assessments of their own weight or BMI. **For program evaluation, self-report is an acceptable proxy for direct measurement of BMI**, though direct measures are recommended for research studies and/or by using correction equations for self-reported BMI as presented.
**Ranjit et al.**[26] Socioeconomic inequalities in children’s diet: the role of the home food environment.	**The home environment matters** for obesity prevention with young people, even when controlling for socio-economic status or among low-income populations. Having regular meals and healthy foods available at home, for example, are important to preventing or reducing childhood obesity.
**Carey et al.**[5] Educational outcomes associated with childhood obesity in the United States: Cross-sectional results from the 2011–2012 National Survey of Children’s Health.	The school environment plays a very important role in obesity prevention. Young people who are overweight or obese may have other health problems and poor educational outcomes. These problems occur early in life, and so **providing extra support to positively influence school educational outcomes** may be critical to preventing longer-term negative social outcomes among those who are obese.
**Evans et al.**[27] Increasing access to healthful foods: A qualitative study with residents of low-income communities.	There is a broad interest in having access to healthy foods in low-income neighborhoods. A consistently reported concern is the **need for large grocery stores with ample produce and grocery selection nearby**. Other approaches to increasing access to healthy foods, such as community gardens, were secondary to having access to a large grocery store.
**Vandewater et al.**[28] Time with friends and physical activity as mechanisms linking obesity and television viewing among youth.	Time spent watching television (TV) has been associated with childhood obesity. However, prevention approaches may need to do much more than limit TV time. The relationship between overweight and TV viewing time may be mediated by social isolation and marginalization. So, **a more comprehensive approach that seeks to reduce social isolation such as prevention of weight bullying and acceptance of a range of body types** among young people may be as important as getting kids away from the TV screen.
**Taber et al.**[29] The association between state bans on soda only and adolescent substitution with other sugar-sweetened beverages: a cross-sectional study.	School policies restricting access to sugar-sweetened beverages may result in the unintended consequence of increases in other vended drinks with added sugars. **States and schools need to have clear, consistent and strict policies around sugar-sweetened beverages** so that if sodas are not allowed at school, then neither are other drinks that contain sugar (e.g., energy drinks). Providing only healthy no- or low-calorie beverage options, or low-fat milk, in schools seems an important step in making our schools healthy for kids.
**Springer et al.**[30] School-level economic disadvantage and obesity in middle school children in central Texas, USA: a cross-sectional study.	**Schools that are economically disadvantaged should be an important target for community prevention efforts** since those schools are likely to have more obese children, independent of individual family socio-economic status. Socioeconomically disadvantaged schools should receive priority for prevention programs and policies related to obesity.

## Conclusions

The Michael and Susan Dell Center for Healthy Living is a unique public-private partnership designed to advance the science around child health, with a particular focus on childhood obesity. Key characteristics of the Center serve to extend existing examples of such partnerships; in addition, the organization as a public-private partnership and its unifying conceptual framework has helped advance important knowledge in this area. It is our intent, through the articles in this special issue, to add to the international dialogue on childhood obesity, nutrition and physical activity, and to contribute scientifically to this important literature. We offer some additional perspectives and data to this dialogue, which we hope may stimulate further research in this critical area of childhood obesity.

## List of abbreviations used

BMI = Body Mass Index; kg = kilogram; m = meter; SCT = Social Cognitive Theory; SSB = Sugar-sweetened beverage; PE = Physical Education.

## Competing interests

Drs. Perry and Hoelscher are founding members of the Michael & Susan Dell Center for Health Living at the University of Texas, School of Public Health Austin Regional Campus. Dr. Kohl serves on the Executive Committee of the Center. Dr. Hoelscher is the Director of the Center. None of the authors have a financial interest in the Center or the Foundation.

## Authors’ contributions

CLP wrote the entire draft of the paper. DMH and HWK edited the paper and helped to direct the review of the literatures. All authors read and approved the final version.

## Declarations

This article has been published as part of *International Journal of Behavioral Nutrition and Physical Activity* Volume 12 Supplement 1, 2015: The science of childhood obesity: an individual to societal framework. The full contents of the supplement are available online at http://www.ijbnpa.org/supplements/12/S1. Publication costs for this supplement were funded by the Michael & Susan Dell Foundation.
